# Loss of Panx1 Impairs Mammary Gland Development at Lactation: Implications for Breast Tumorigenesis

**DOI:** 10.1371/journal.pone.0154162

**Published:** 2016-04-21

**Authors:** Michael K. G. Stewart, Isabelle Plante, Silvia Penuela, Dale W. Laird

**Affiliations:** 1 Department of Physiology and Pharmacology, University of Western Ontario, 1151 Richmond Street, London, Ontario, N6A 5C1, Canada; 2 Centre INRS–Institut Armand-Frappier, 531 Boulevard des Prairies, Laval, Québec, H7V 1B7, Canada; 3 Department of Anatomy and Cell Biology, University of Western Ontario, 1151 Richmond Street, London, Ontario, N6A 5C1, Canada; Emory University School of Medicine, UNITED STATES

## Abstract

Pannexin1 (Panx1) subunits oligomerize to form large-pore channels between the intracellular and extracellular milieu that have been shown to regulate proliferation, differentiation and cell death mechanisms. These key cellular responses are ultimately necessary for normal tissue development and function but the role of Panx1 in development, differentiation and function in many tissues remains unexplored, including that of the breast. Panx1 was identified to be expressed in the mammary gland through western blot and immunofluorescent analysis and is dynamically upregulated during pregnancy and lactation. In order to evaluate the role of Panx1 in the context of mammary gland development and function, *Panx1*^*-/-*^ mice were evaluated in comparison to wild-type mice in the mammary glands of virgin, lactating and involuting mice. Our results revealed that Panx1 ablation did not affect virgin or involuting mammary glands following histological and whole mount analysis. Panx1 was necessary for timely alveolar development during early lactation based on a decreased number of alveolar lumen following histological analysis and reduced proliferation following Ki67 immunofluorescent labelling. Importantly, the loss of Panx1 in lactating mammary glands did not overtly affect epithelial or secretory differentiation of the mammary gland suggesting that Panx1 is not critical in normal mammary gland function. In addition, PANX1 mRNA expression was correlated with negative clinical outcomes in patients with breast cancer using *in silico* arrays. Together, our results suggest that Panx1 is necessary for timely alveolar development following the transition from pregnancy to lactation, which may have implications extending to patients with breast cancer.

## Introduction

Mammary gland development is a dynamic process occurring mostly after birth [1]. The mouse mammary gland undergoes extensive gland remodeling through two main phases of development following the onset of puberty and pregnancy [2]. During puberty, epithelial ductal elongation and branching loosely invades the adipocyte-rich mammary stroma [3]. The mammary gland undergoes terminal differentiation following the onset of pregnancy characterized by extensive proliferation and lobuloalveolar differentiation as numerous alveoli fill the mammary gland for secretory function during lactation [2]. Following weaning of pups, the mammary gland reverts back to a pre-pregnant state in a process known as involution [4]. These processes require extensive control of proliferation, differentiation, invasion, and cell death mechanisms mediated by hormonal signaling, local epithelial-stromal interactions and direct cell-cell communication mediated by gap junctions [1,5].

While the roles of the mammary gap junction proteins Cx43, Cx26, Cx30 and Cx32 are beginning to be defined within the mammary gland, particularly through the use of genetically-modified mice, less is known about the large-pore channel proteins pannexins in the context of the mammary gland [6]. Pannexins, similar to connexin hemichannels, oligomerize to form large protein-lined pores capable of transferring ions and metabolites, such as ATP and Ca^2+^, between the intracellular and extracellular milieu [7,8]. However, unlike connexin hemichannels, pannexin channels are glycosylated, insensitive to physiological levels of extracellular Ca^2+^ and can be opened at normal resting membrane potentials [9–11]. As a result, this suggests that pannexins have unique functions within tissues that warrants further investigation.

Three members of the pannexin family have been described in the mammalian genome, each predicted to have a similar topology to the vertebrate gap junction proteins connexins [7,12]. Due to its ubiquitous expression, pannexin1 (Panx1) is the best characterized and has been identified in both rodent and human organs that include the brain, muscle, and skin [13–16], as well as many other tissues including the mouse mammary gland and human breast as noted in NCBI’s gene expression Omnibus database (1416379 ID, ID 49755742, [17]). Panx1 channels can be activated and opened by multiple stimuli that may occur during mammary gland development and remodeling, including mechanical stimulation, caspase cleavage, intracellular Ca^2+^, and extracellular ATP [4,18–23].

Panx1 has also been shown to be dynamically regulated during brain, muscle and skin development [14–16]. Panx1 has been associated with changes in migration of primary keratinocytes, proliferation of dermal fibroblasts, neural stem cells and neural progenitor cells, as well as differentiation of skeletal muscle myoblasts [15,24,25]. Importantly, all of these cellular processes are necessary for normal mammary gland development and function suggesting a role for Panx1 in the highly regulated mammary gland [1]. In addition, Panx1 channels were shown to mediate the release of ATP from apoptotic cells which acts to recruit phagocytes for cell clearance following Panx1 C-terminal cleavage by caspases [19]. This is intriguing as macrophages have been shown to be important during mammary gland involution [26]. With the developmental and physiological roles of Panx1 are beginning to be elucidated, it is not surprising that Panx1 has been implicated in many pathologies, including tumorigenesis (as reviewed by Penuela et al. [27]).

Breast cancer is the most severe pathology associated with the breast and is the leading cause of cancer mortality in women worldwide [28]. Recently, Panx1 was shown to be mutated in metastatic breast cancer cell lines, leading to increased ATP-channel activity and promotion of breast cancer cell survival during extravasation [29]. This may suggest that Panx1 functions as a tumor facilitator in breast cancer similar to that described in melanoma, albeit through different reported mechanisms [29,30]. However, as Panx1 has also been suggested to act as a tumor suppressor in gliomas, and squamous and basal cell carcinomas, the role of Panx1 may be dependent on the type of cancer or stage of disease [31,32]. As tumors frequently exploit signaling pathways critical in organ morphogenesis, we set out to evaluate the role of Panx1 in normal mammary gland development to increase our understanding of how the role of Panx1 may be dysregulated in tumorigenesis. In addition, we explored potential implications of Panx1 as a biomarker in breast cancer.

## Materials and Methods

### Animals

All experiments were approved by the Animal Care Committee at Western University and conducted according to the guidelines of the Canadian Council on Animal Care. Panx1^-/-^ mice were generated as previously described [33]. Panx1^-/-^ mice were developed on a homogeneous C57BL/6 background in which Panx1 was systemically ablated. C57BL/6N mice (Panx1^+/+^) acted as control mice for all experiments. Mice were collected at 4 weeks, 7 weeks, and 14–20 weeks at parturition (L0), early lactation (L2) and involution (Forced weaned at L15 and collected I3). Mice were genotyped as previously described [33]. Following sacrifice of mice using CO_2_, body weights were recorded prior to dissection of inguinal and thoracic mammary glands. Four mammary glands were dissected for subsequent paraffin processing, whole mount analysis, cryosections and for protein lysates. Right inguinal mammary gland weights were recorded. For all experiments, at least five different animals per group were evaluated. Panx1 null pup weights were recorded from multiple lactating dams.

### Western blot analysis

Following dissection, mammary gland tissue was frozen at -80°C. Mammary gland tissues were homogenized on ice in lysis buffer (1% Triton X-100, 150 mM NaCl, 10 mM Tris-HCl, pH 7.4, 1 mM EDTA, 0.5% NP-40), supplemented with protease inhibitor mixture (Roche-Applied Sciences) and phosphatase inhibitors (100 mM NaF and 100 mM Na_3_VO_4_). Total protein lysates were quantified using the bicinchronic acid assay similar to the manufacturer’s instructions (Pierce). 60 ug of protein was loaded and resolved using 10% SDS-PAGE and transferred to nitrocellulose membranes using the iBlot Dry Blotting system (Invitrogen). Membranes were blocked using 5% bovine serum albumin (BSA) and 0.05% Tween20 in PBS for 1 hr at room temperature. Membranes were incubated with rabbit anti-Panx1 antibodies targeting the C-terminal (0.4 μg/ml, [9]), goat anti-b -casein (1:1000, sc-17971, Santa Cruz Biotechnology) and rat anti-Hsc70 (1:5000, SPA-815, Stressgen Bioreagents) antibodies diluted in blocking solution at 4°C overnight. Membranes were washed in PBST and subsequently incubated with Alexa 800-conjugated goat anti-rabbit, Alexa 800-conjugated donkey anti-mouse, Alexa 680-conjugated goat anti-rat and Alexa 680-conjugated donkey anti-goat (1:5000, Life Technologies) secondary antibodies followed by visualization and quantification using the Odyssey Infrared Imaging System (Li-Cor Biosciences). N = 3. HEK 293T cells were used a negative control and HEK 293T cells overexpressing Panx1 were used as a positive control.

### Real-time PCR analysis

Total RNA was extracted using the Qiagen RNeasy kits (Qiagen) from the mammary gland of 7-week-old females Panx1^-/-^ and Panx1^+/+^ mice. cDNA was generated using the RevertAid H minus, first-strand cDNA synthesis kit (Fermentas). Panx1 transcript levels were determined using mouse Panx1-specific primers (5' ACAGGCTGCCTTTGTGGATTCA3'; 5' GGGCAGGTACAGGAGTATG3') and the iQ SYBR green Supermix (Bio-Rad, Mississauga) in a Bio-Rad CFX96 real-time system. Results were normalized to β-2 microglobulin (5'CCCACTGAGACTGATACATACGC3'; 5' GGTTCAAATGAATCTTCAGAGCAT 3'). N = 3.

### Immunofluorescence microscopy

Paraffin embedded sections (6 um) were deparaffinised in xylene and rehydrated in descending concentrations of ethanol before being washed in ddH_2_O. Sections underwent antigen retrieval using Vector Antigen Unmasking Solution (Vector Labs) by microwaving them for 5 mins at 80% power. Sections were allowed to cool for 15 mins prior to being rinsed in PBS and placed in a sub-boiling second antigen retrieval solution (10 mM Tris Base, 1 mM EDTA (pH 9.0) for 30 min prior to being rinsed in PBS. Crysections were sectioned (7 um), stored in -80°C and subsequently fixed in 10% neutral buffered formalin and rinsed in PBS. Sections were then blocked (3% BSA and 0.2% Triton X-100 in PBS) for 1 hr at room temperature. The following primary antibodies were incubated on samples diluted in blocking solution overnight at 4°C; rabbit anti-Panx1 (4 ug/ml, or with peptide pre-adsoption assays as previously described [9]), mouse anti-cytokeratin 14 (1:300, Neomarkers, CL002), mouse anti-pan-cytokeratin (1:400, Abcam, ab7753), rabbit anti-periplipin (1:400, Cell Signaling, 9349), rabbit anti-Ki67 (1:400, Abcam, ab66155), anti-cleaved caspase 3 (1:400, Cell Signaling, D175), rabbit anti-keratin8 (1:400, Abcam, ab53280), anti-E-cadherin (1:400, BD Transduction Laboratories, 610182), anti-b-catenin (1:400, BD Transduction Laboratories, 610154), anti-Cx26 (Cryosections, 1:100, Invitrogen, 51–2800), anti-Cx30 (Cryosections, 1:100, Invitrogen, 71–2200), anti-Cx32 (Cryosections, 1:100, Sigma, C3470) and anti-β-casein (1:400, Santa Cruz Biotechnology, sc-17971). Primary antibodies were visualized by incubating sections with Alexa Fluor^®^ 555-conjugated anti-rabbit, anti-mouse or anti-goat (1:400, Molecular Probes, A21425, A21429 or A21431) and Alexa Fluor^®^ 488-conjugated anti-rabbit or anti-mouse (1:400 dilution, Molecular Probes, A11008 or A11017) secondary antibodies for 1 hr at room temperature. Hoechst stain was used to visualize nuclei before being mounted using Airvol. Immunolabeled sections were imaged (5–10 images per sample) using a Leica DM IRE2 inverted epifluorescence microscope equipped with Velocity 6.3.0 imaging software. For cytokeratin area quantification, green only fluorescent images were converted to binary using ImageJ and the pixel area was measured per 0.3mm^3^. For adipocyte quantification, perilipin-positive cells were counted per 0.3mm^3^. For Ki67 and connexin plaque quantification, the number of Ki67 positive cells/connexin plaques was quantified per 0.3mm^3^. In addition, the blue only fluorescent images were converted to binary using ImageJ and the pixel area was measured per 0.3mm^3^. Graphs represent the mean ratio of the number of Ki67 positive cells or connexin plaques to blue fluorescent pixel area (nuclei). N≥5.

### Whole mount analysis

Whole mount analysis was performed similar to that described in Plante et al. 2011 [34]. Briefly, mammary glands were dissected, flattened out on slides, and submersed in Carnoy’s fixative for 4 hrs at room temperature or overnight at 4°C. Glands were then submersed in 70% ethanol and gradually rehydrated in ddH_2_O before being stained overnight in carmine alum stain at room temperature. Glands were then gradually dehydrated (ddH_2_O, 70%, 95%, 100%, xylene; 5 min) and stored in methyl salicylate. Whole mounts were captured using a stereoscopic Sony camera on a light board. Virgin ductal elongation was quantified using calipers as previously described [35,36] by measuring the ratio of the distance from the bottom of the lymph node to the end of the longest duct relative to the distance from the bottom of the lymph node to the edge of the fat pad.

### Histology

Paraffin blocks were sectioned (6 um), deparaffinized in xylene (10 min) and gradually rehydrated in ethanol (100%, 95%, 70%; 5 min) prior to submersion in Harris’ hematoxylin for 2 min. Slides were washed, dipped in acid ethanol (4X) and placed in 70% ethanol (1 min) prior to submersion in eosin (1 ml of acetic acid in 250 ml of eosin; 2 min). Slides were gradually dehydrated (70%, 95%, 100%, xylene; 1 min) and mounted using cytoseal (Richard-Allan Scientific). Histological analysis was performed by imaging 5–10 arbitrary images throughout the entire mammary gland using a 5X and 40X objective lens and a ProgRes C5 camera (Jenoptik) and ProgRes Mac CapturePro 2.7.6 imaging software. The average number of alveolar lumens was quantified, with the mutant and control mouse mammary glands blinded to the investigator, using ImageJ software. In addition, the average alveolar lumen area was quantified using ImageJ in which the length and width (pixels) of alveolar lumens was measured and the average area of the alveolar lumen was estimated by calculating the elliptical area. N≥5.

### Evaluation of Panx1 mRNA *in silico*

Using the publicly available Kaplan-Meier Plotter (http://kmplot.com) described by Gyorffy et al. 2010 [37], we compared high and low mRNA expression groups of PANX1 (Affy id 204715_at) in human breast cancer samples to clinical endpoints using the HGU133A and HGU133 Plus 2.0 microarrays as previously described [38]. Our analysis was performed on the 2014 version of the database that included data from 4142 patients from all grades of breast cancer. We set our parameters to remove redundant samples, exclude biased arrays, auto-select for best cutoff and to use only the JetSet best probe set. PANX1 was evaluated in relation to overall survival (OS) (n = 1117), relapse-free survival (RFS) (n = 3554), distant metastasis free survival (DMFS) (n = 1609), as well as evaluating Panx1 in relation to OS and RFS in distinct subgroups such as by molecular subtype including Luminal A (ESR1+/HER2-/MKI67low; n = 504), Luminal B (ESR1+/HER2-/MKI67high and ESR1+/HER2+; n = 320), Basal (ESR1-/HER2-; n = 204) and HER2 (ESR1-/HER2+; n = 89) or lymph node positive patient samples (n = 945).

### Statistical analysis

All statistical analyses of mouse studies were performed using GraphPad Prism 4.03 software in which statistical analysis compared means using a two-tailed unpaired student t-test. A two-way ANOVA was performed on pup weights. Error bars represented ± SEM. For assessment of Panx1 as a biomarker, a log-rank test was performed using the online tool as described [39]. For all experiments, a p value of less than 0.05 was considered significant.

## Results

### Panx1 is dynamically regulated throughout mammary gland development

**Fig 1:**
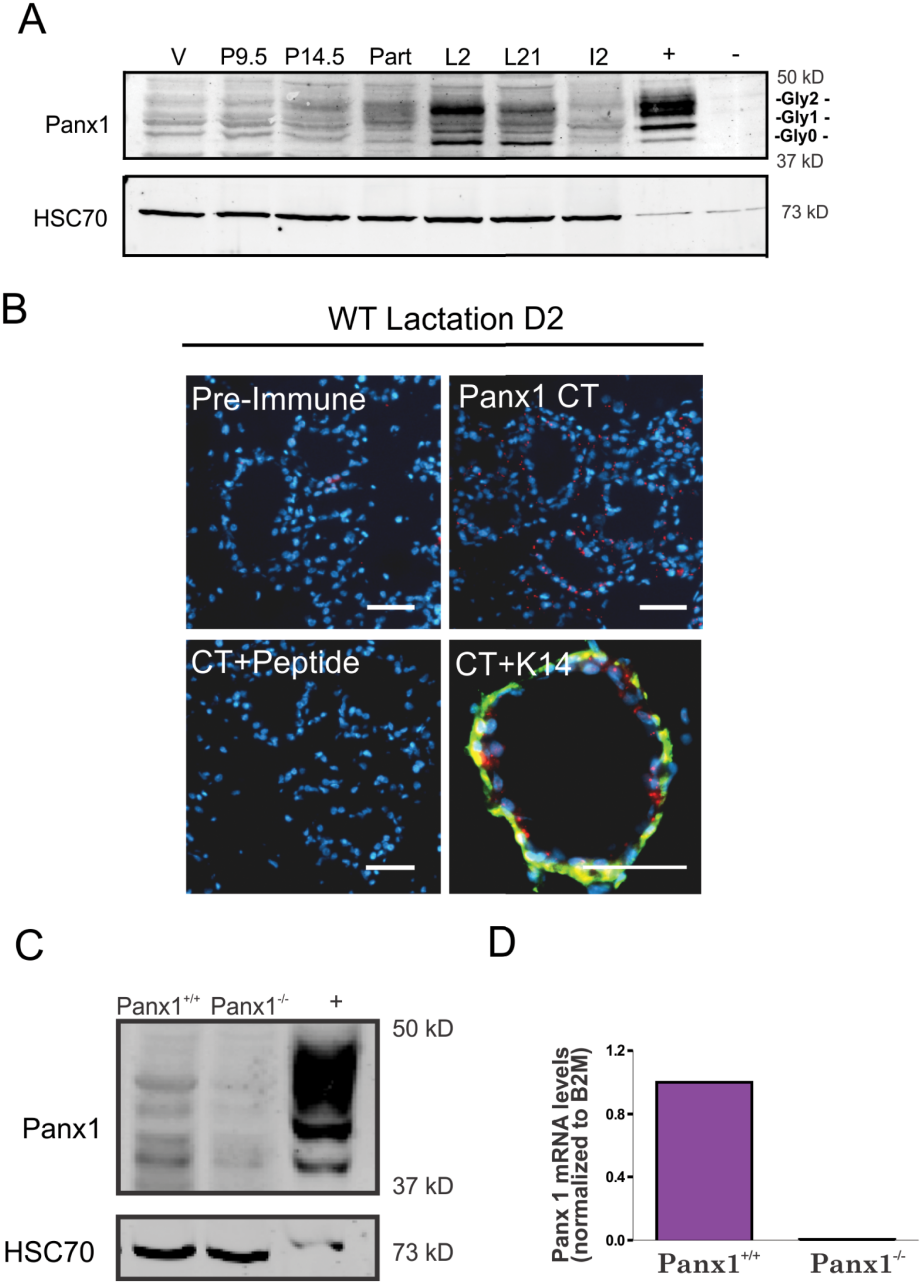
Panx1 is dynamically regulated in the mammary gland and ablated in Panx1^-/-^ mice. (A) Western blot analysis revealed that Panx1 is upregulated following the onset of pregnancy and remains elevated throughout lactation. Multiple bands on the western blot represent various Panx1 species due to changes in glycosylation (Gly0, Gly1 and Gly2). (B) Punctate staining observed for Panx1 (red) is not observed using pre-immune serum or following adsorption of the antibody with cognate peptide and does not significantly colocalize with the myoepithelial marker keratin14 (K14, green). Hoescht (blue) denotes nuclei. Scale bar = 50 um. (C, D) Western blot and real-time PCR analysis revealed that Panx1 is not expressed in Panx1^-/-^ compared to Panx1^+/+^ mice.—represents HEK 293T cells and + represents these cells overexpressing Panx1. Values are means ± S.E.M. For all experiments, N = 3.

Mammary gland lysates from wild-type 7 week old virgin, and 14–20 week old pregnant, lactating and involuting Panx1^+/+^ mice were assessed by western blot for the expression of Panx1. Panx1 immunoblots revealed the multiple glycosylated species of Panx1 (Gly0, Gly1 and Gly2) [8,9], at all stages of development and that Panx1 is upregulated in pregnant and lactating mice (Fig 1A). As Panx1 levels appeared to peak during early lactation, mammary gland sections of Panx1^+/+^ mice at lactation D2 were immunolabeled for Panx1 (with or without cognate peptide) to reveal its localization in the mammary gland (Fig 1B). Panx1 was localized to mainly luminal epithelial cells, based on the reduced frequency of detectable co-localization with the myoepithelial marker keratin14 (Fig 1B). Mammary glands from Panx1^-/-^ and Panx1^+/+^ mice at lactation were further evaluated for Panx1 protein and mRNA expression using western blot and real-time PCR analysis, respectively. Panx1 was found to be ablated from the mammary glands of Panx1 null mice (Fig 1C and 1D). Collectively, these studies revealed that Panx1 is dynamically expressed in the pregnant mammary gland and ablated in Panx1^-/-^ mouse mammary glands.

### Virgin Panx1^-/-^ mice retain normal mammary glands

**Fig 2:**
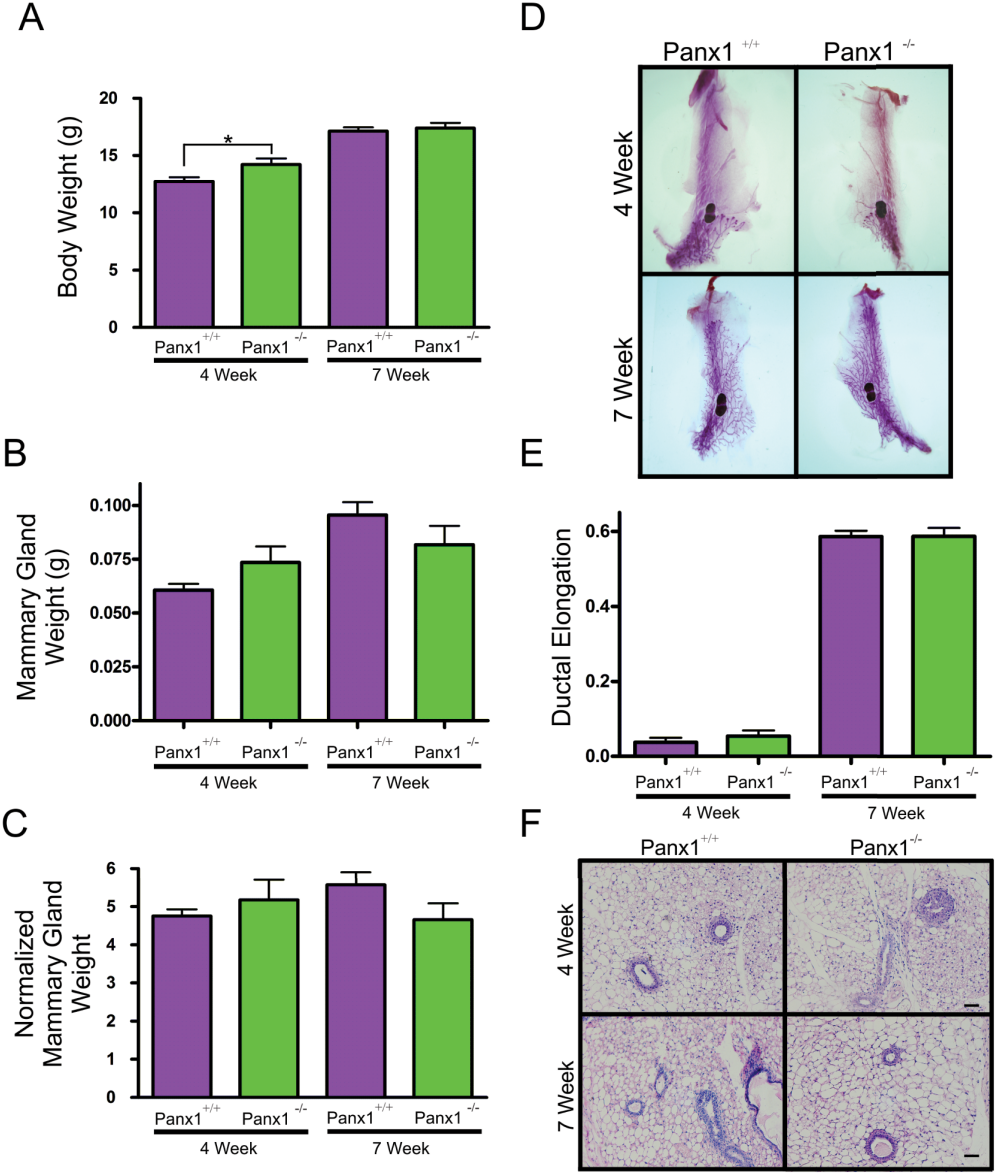
Virgin Panx1^-/-^ mice have normal mammary glands. (A) Evaluation of body weights at 4 and 7 weeks revealed that pre-pubertal Panx1^-/-^ mice were significantly larger at 4 weeks compared to control mice. Values are mean body weights ± S.E.M. * P <0.05. (B, C) Mammary gland weight and normalized mammary gland weight were not significantly different in Panx1^-/-^ compared to Panx1^+/+^ mice. Values are mean weights ± S.E.M. (D) Whole mount analysis with carmine alum staining revealed normal epithelial ductal architecture embedded in a well-developed stroma. (E) Quantification of ductal elongation revealed no significant differences in knockout and control mice. Values are mean lengths ± SEM. (F) Histological evaluation of hematoxylin and eosin stained glands revealed normal tissue architecture in virgin Panx1^-/-^ compared to control mice. N = 9. Scale bar = 50 um.

To evaluate how Panx1 ablation affected ductal development of the virgin mammary gland following the onset of puberty, mammary glands from 4 and 7 week old mice were weighed and subjected to whole mount and histological analysis. Although body weights were significantly elevated in 4 week old Panx1^-/-^ mice (Fig 2A), this did not correspond to an increased mammary gland weight (Fig 2B and 2C). Body weight and normalized mammary gland weight were similar in 7 week old mice compared to controls (Fig 2A–2C). Whole mount analysis of 4 week old mice revealed a rudimentary ductal structure in the mammary glands of Panx1 knockout mice similar to control mice suggesting that loss of Panx1 does not significantly impede embryonic mammary gland development (Fig 2D). In addition, the relative duct length of 4 and 7 week old whole mounts from Panx1^-/-^ mice was similar to control mice (Fig 2E). Similar to whole mount analysis, Panx1 knockout mice had comparatively normal histology as epithelial ducts were embedded within a well-developed mammary fat pad in Panx1 null and control mice (Fig 2F). Taken together, virgin mammary glands from Panx1 knockout mice develop similar to wild-type mice.

### Panx1^-/-^ mice at parturition have normal mammary glands

**Fig 3:**
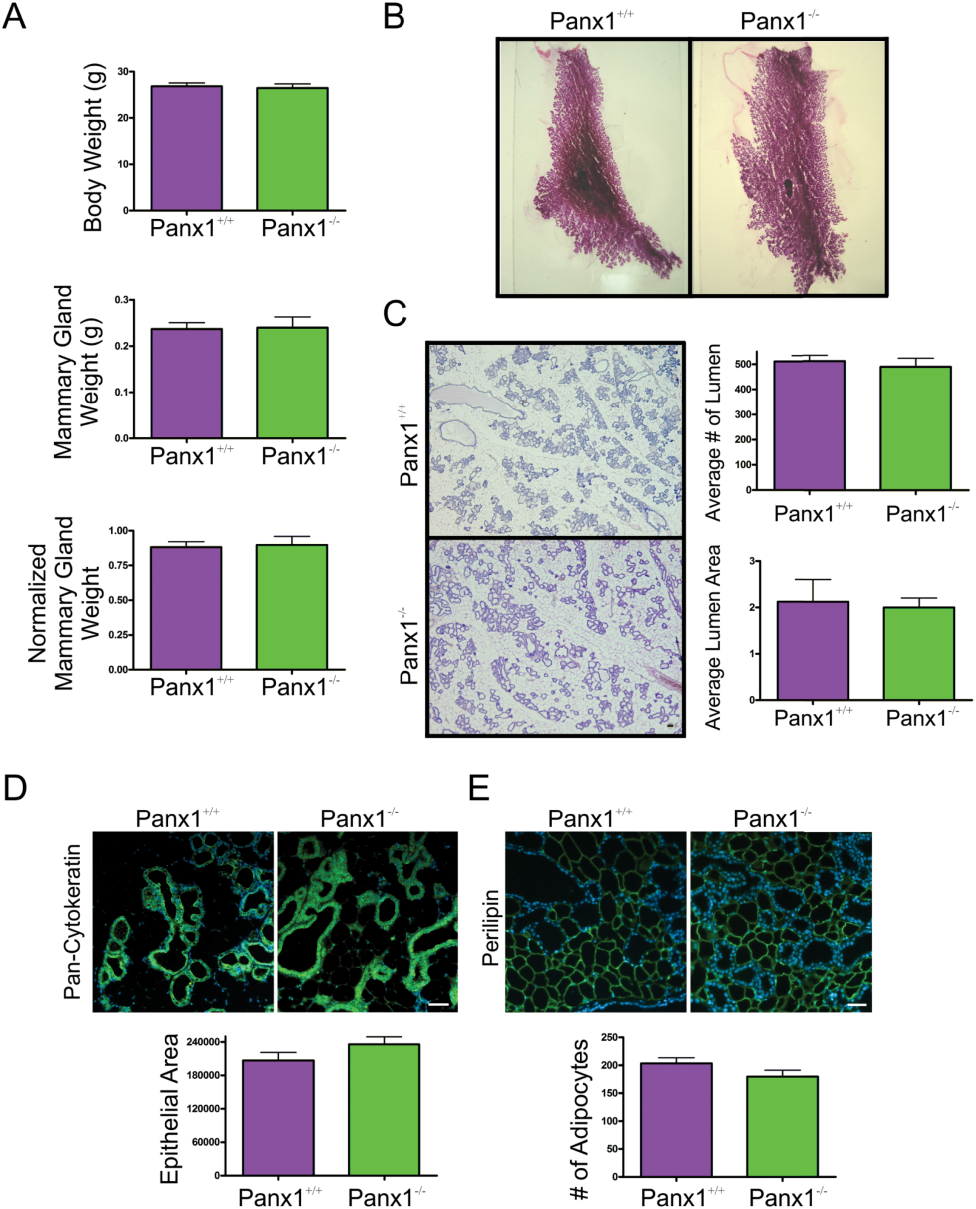
Panx1^-/-^ mice have normal mammary glands at parturition. (A) Evaluation of body weight, mammary gland weight and normalized mammary gland weight revealed that Panx1^-/-^ mice were similar to control mice at parturition. (B) Whole mount analysis revealed numerous alveoli filling the mammary fat pad. (C) Histological evaluation of haematoxylin and eosin stained glands revealed normal tissue architecture, a similar average number of alveoli and a similar average alveolar lumen area in Panx1 null mice compared to control mice. (D) Quantification of the average epithelial area as assessed with immunofluorescent analysis using pan-cytokeratin (green) revealed similar epithelial area in the mammary gland of Panx1^-/-^ compared with control mice. (E) Quantification of the average number of adipocytes as assessed with immunofluorescent analysis using perilipin (green) revealed a similar number of adipocytes in Panx1^-/-^ mice compared with control mice. Hoescht (blue) denotes nuclei. Bars are means ±S.E.M. N = 6. Scale bars = 50 um.

In order to assess the role of Panx1 following pregnancy, mammary glands from Panx1^-/-^ mice and wild-type control mice were collected at parturition, weighed, and assessed for changes in gland architecture using histological and whole mount approaches. Body weights, mammary gland weight and normalized mammary gland weight were similar between Panx1^-/-^ and control mice (Fig 3A). Whole mount and histological analysis revealed similar tissue architecture of Panx1^-/-^ mice compared to control mice (Fig 3C). Quantification of the average number and area of the alveolar lumen between Panx1 knockout and wild-type H&E stained sections was similar suggesting comparable alveolar development at parturition (Fig 3C). Similarly, quantification of the pixel area of the epithelial marker pan-cytokeratin in mammary gland sections of Panx1^-/-^ and Panx1^+/+^ mice revealed similar epithelial area (Fig 3D). Finally, immunofluorescent labelling and quantification of the number of adipocytes revealed similar stromal development in the glands of Panx1 knockout and wild-type mice (Fig 3E).

### Panx1^-/-^ Mice at Lactation have Impaired Alveolar Development

**Fig 4:**
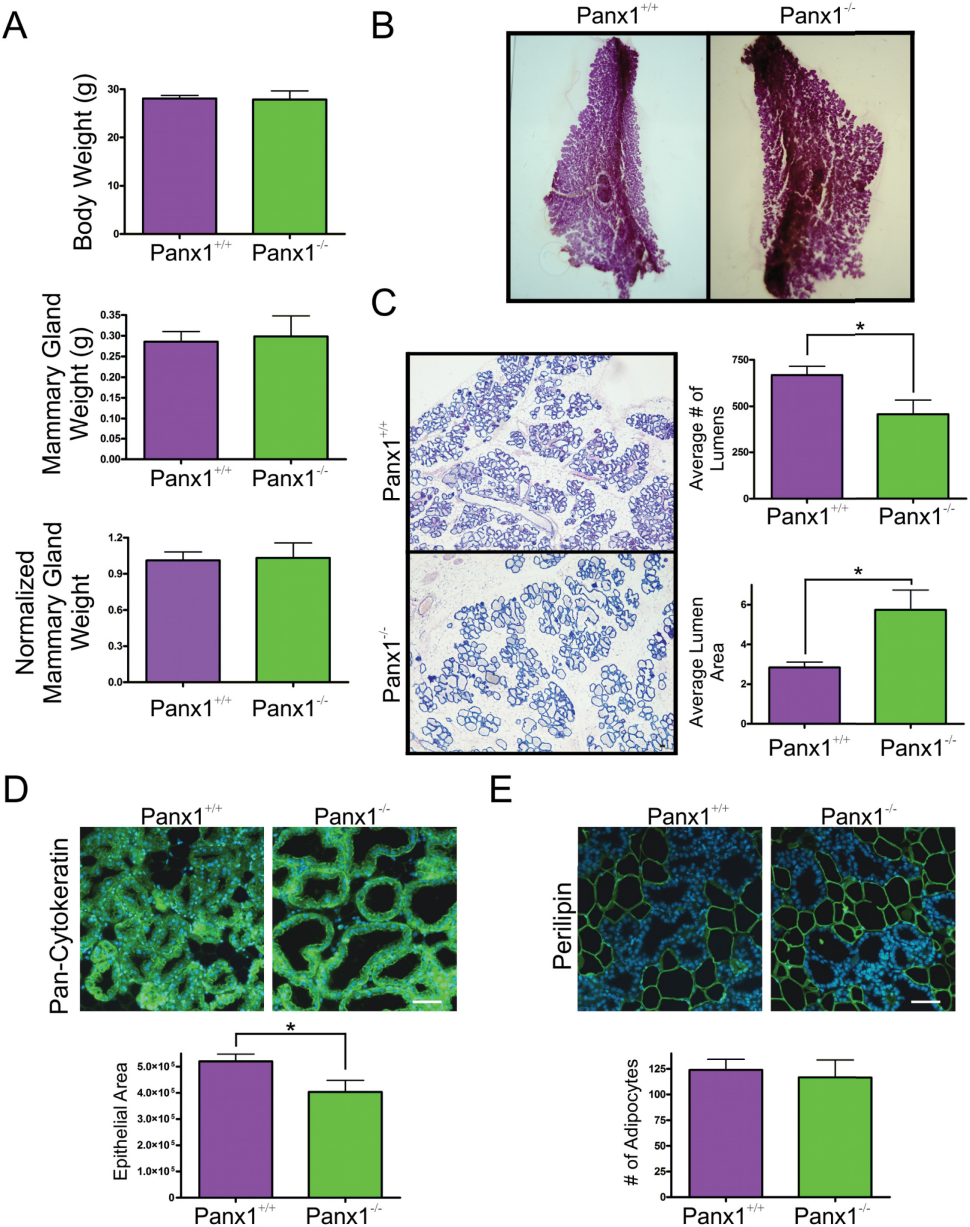
Panx1^-/-^ mice present with a reduction in the number of alveoli during early lactation. (A) Evaluation of body weight, mammary gland weight and normalized mammary gland weight revealed that Panx1^-/-^ mice were similar to control mice. (B) Whole mount analysis revealed numerous alveoli filling the mammary fat pad. (C) Histological evaluation of hematoxylin and eosin stained glands revealed a reduction in the number of alveoli in the mammary glands of Panx1^-/-^ mice that were significantly larger than those from Panx1^+/+^ mice. (D) Quantification of the average epithelial area as assessed with pan-cytokeratin (green) revealed a significant decrease in the amount of epithelium in the mammary gland of Panx1^-/-^ mice compared with control mice. (E) Quantification of the average number of adipocytes, as assessed with perilipin (green), revealed similar cell numbers in the mammary gland of Panx1^-/-^ mice compared with control mice. Hoescht (blue) denotes nuclei. Values are means ± SEM. N = 6. Scale bars = 50 um.

To assess whether differences in gland development occurred following feeding of the pups, mammary glands of Panx1^-/-^ and Panx1^+/+^ mice were assessed 48 hours after feeding and compared to Panx1^+/+^ mice. Similar to mice on the day of parturition, body weights, mammary gland weights and normalized mammary gland weights were not significantly different during early lactation in Panx1^-/-^ and control mice (Fig 4A). Interestingly, whole mount and histological analysis of mammary glands revealed a significant decrease in the average number of alveolar lumens of Panx1 knockout mice with a significant increase in the average alveolar lumen area compared to wild-type mice suggesting impaired alveolar development in early lactation (Fig 4B and 4C). Furthermore, a reduction in the average epithelial area, but not in the number of adipocytes glands, of Panx1^-/-^ mice compared to Panx1^+/+^ mice supported a role for impaired alveolar development of early lactating Panx1 knockout mice (Fig 4D and 4E).

**Fig 5:**
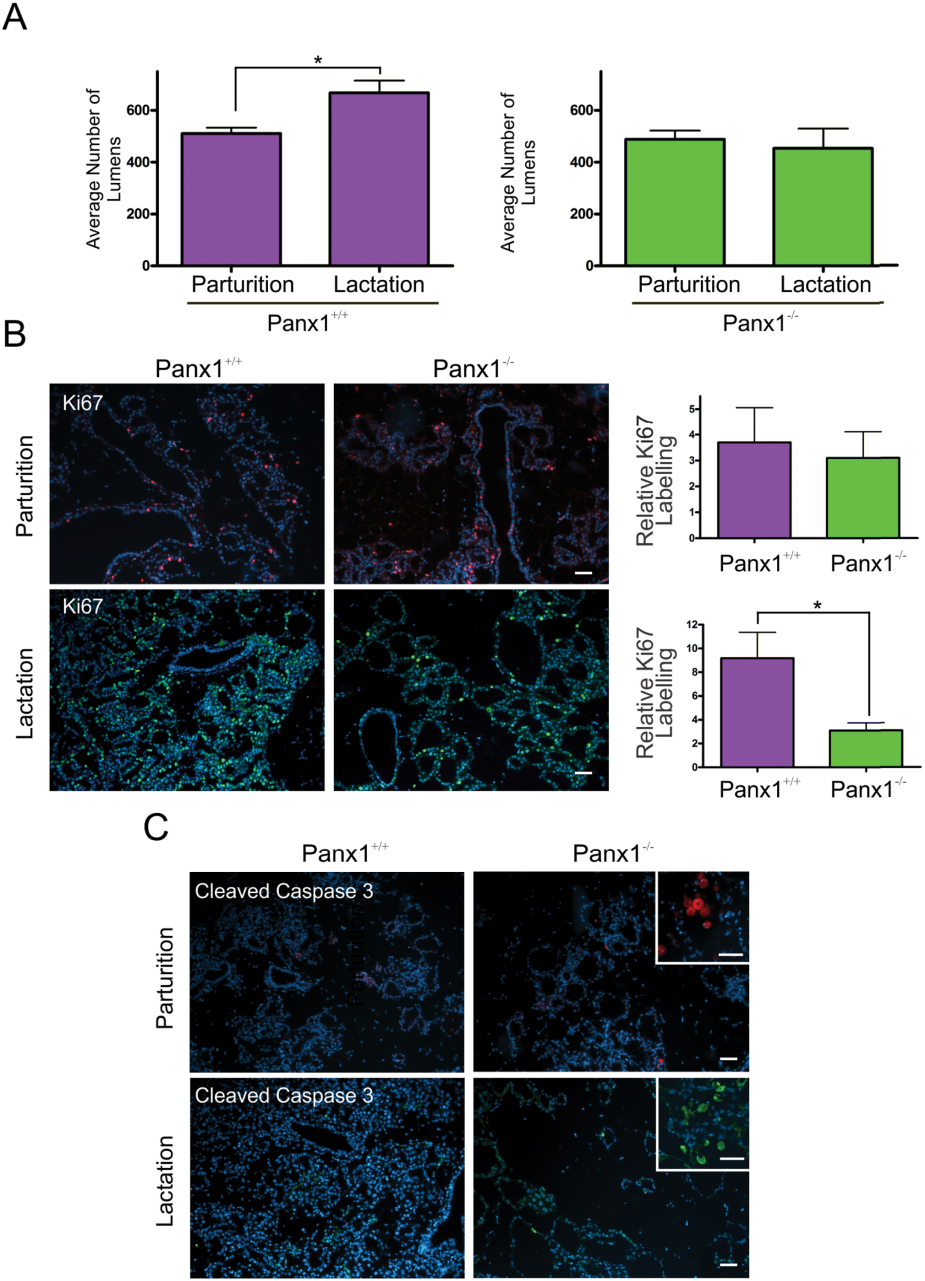
Panx1^-/-^ mice have reduced cell proliferation during early lactation. (A) The average number of alveolar lumens was significantly increased in early lactation compared to parturition in Panx1^+/+^ mice which was not observed in Panx1^-/-^ mice. (B) Immunofluorescent analysis of the proliferation marker Ki67 (Red; parturition, green; lactation) revealed significantly reduced proliferation in mammary glands of Panx1^-/-^ mice compared to controls during early lactation but not at parturition. (C) Immunofluorescent analysis of the apoptotic marker cleaved caspase3 revealed little apoptosis in the glands of knockout mice, similar to controls. Inserts represent positive controls in the involuting mammary gland. Hoescht (blue) denotes nuclei. N = 6. Scale bars = 50 um.

Comparison of the number of alveolar lumens in mammary glands at parturition and early lactation revealed a significant increase in the number of alveoli during the 48 hours following parturition in Panx1^+/+^ mice but not in Panx1^-/-^ mice (Fig 5A). To assess whether this difference was the result of impaired proliferation or increased apoptosis, mammary glands of Panx1 knockout and wild-type mice were immunolabelled with the proliferation marker Ki67. This study revealed a significant decrease in the number of Ki67 positive cells during early lactation, but not parturition, between Panx1^-/-^ and control mice (Fig 5B). Qualitative assessment of cleaved caspase-3 immunolabelling revealed relatively few apoptotic cells in the lactating glands of Panx1^-/-^ mice compare to wild-type mice suggesting that impaired alveolar development was the result of impaired proliferation as opposed to increased cell death (Fig 5C).

**Fig 6:**
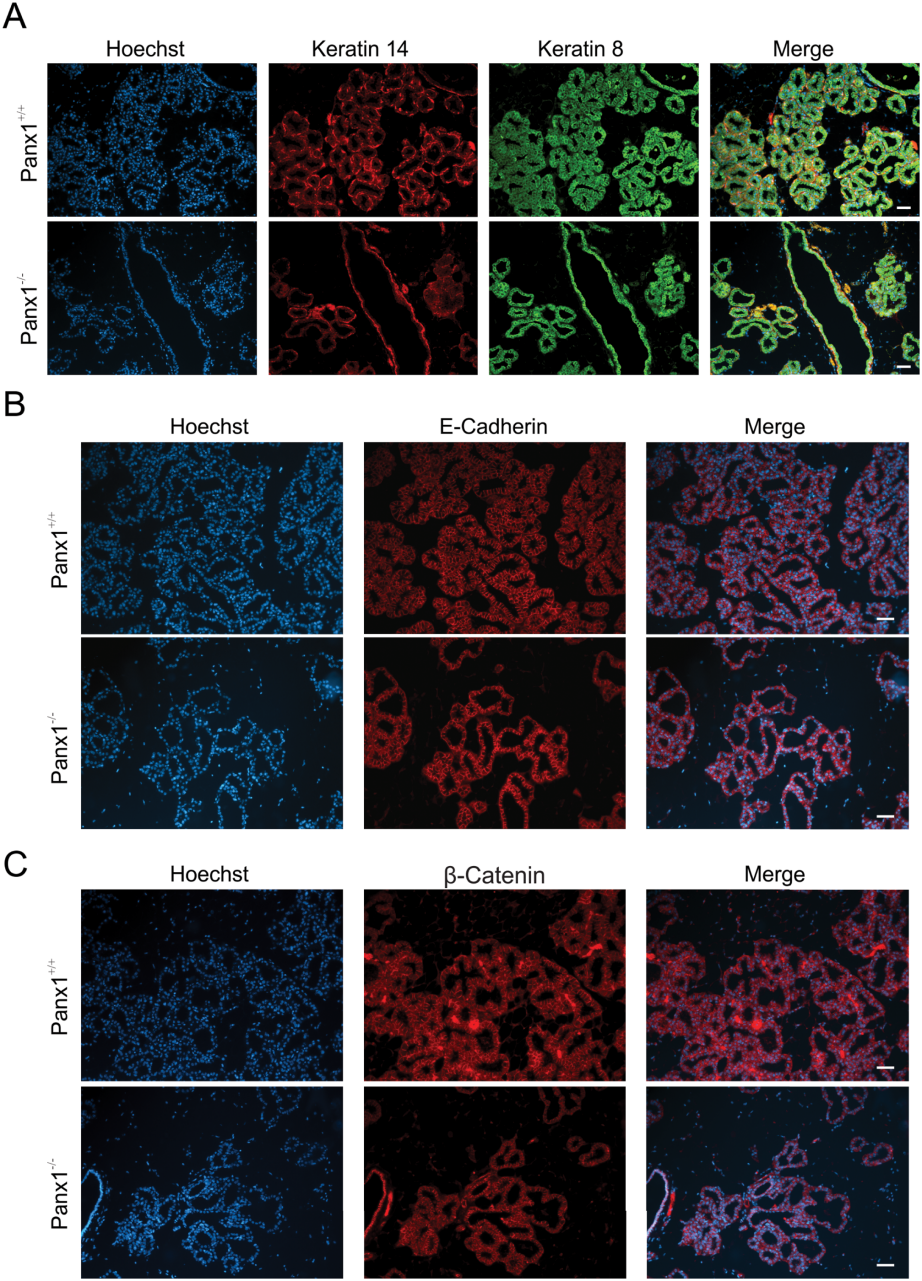
Panx1^-/-^ mice have normal mammary gland epithelial differentiation at lactation. (A) Immunofluorescent analysis of luminal epithelial marker keratin 8 (green) and myoepithelial marker keratin14 (red) revealed a similar staining pattern in Panx1^-/-^ mice compared to control mice during lactation. (B, C) Immunofluorescent analysis of mammary differentiation markers E-cadherin (B, red) and b-Catenin (C, red) revealed a similar staining profile in Panx1^-/-^ mice and Panx1^+/+^ mice. Hoescht (blue) denotes nuclei. N = 6. Scale bars = 50 um.

**Fig 7:**
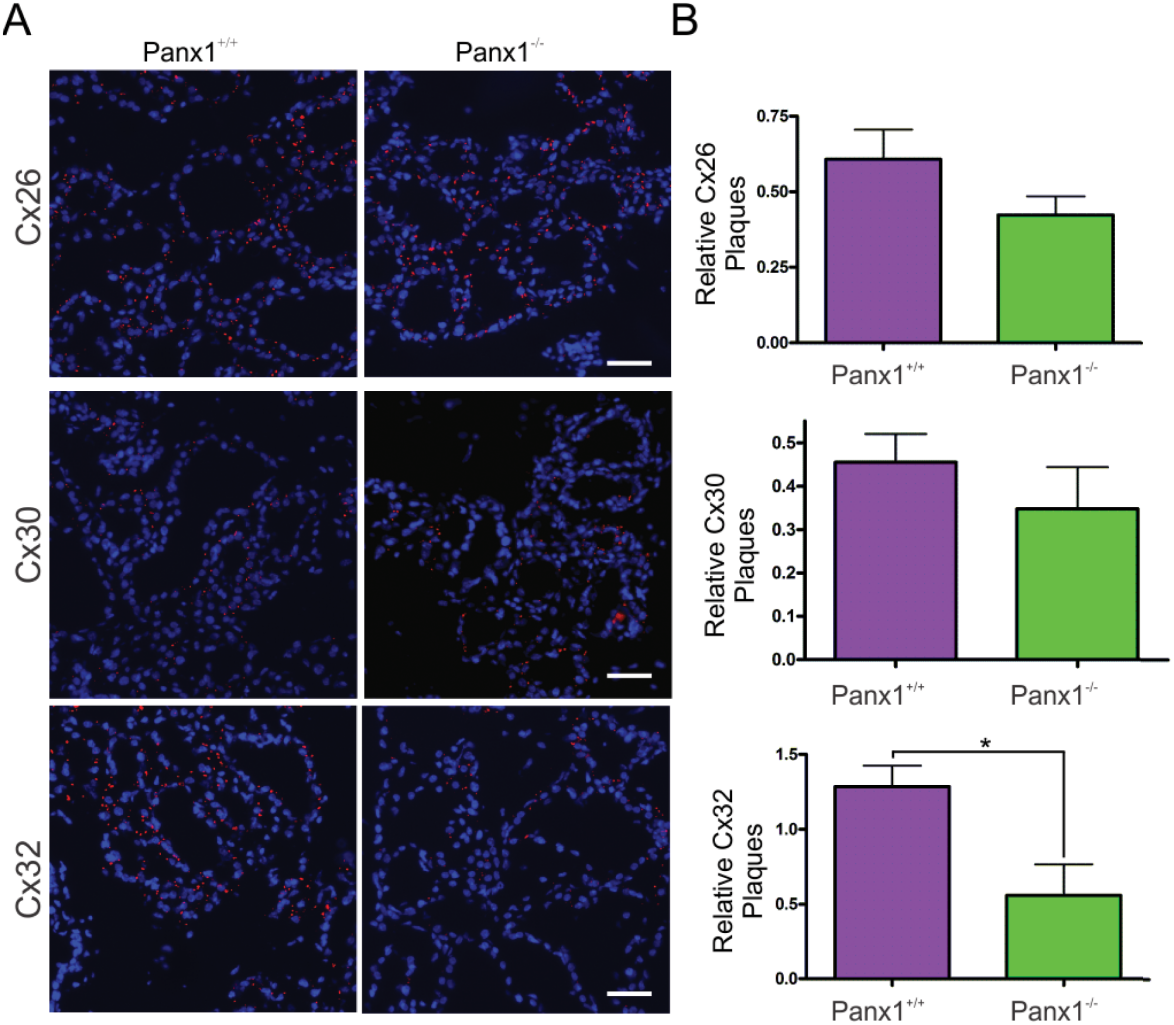
Lactating Panx1^-/-^ mice have fewer Cx32 gap junctions in the mammary gland. (A) Immunofluorescent analysis of mammary gland cryosections during early lactation for Cx26, Cx30 and Cx32 (red) and cytokeratin14 (green) revealed no change in Cx26 and Cx30 gap junctions in knockout mice, while fewer Cx32 gap junctions were observed compared to control mice. Hoescht (blue) denotes nuclei. Scale bar = 50 um. (B) Values represent the mean number of connexin plaques (red) relative to the pixel area of the nuclei (blue), multiplied by a factor of 1x10^2^, per 0.3mm^2^ ± S.E.M. N = 5.

In order to assess whether the defect in alveogenesis affects the differentiation of the mammary glands, markers of differentiation including the luminal markers keratin8, E-cadherin and b-catenin as well as the myoepithelial marker keratin14, were assessed by immunofluorescent analysis in lactating mice (Fig 6). Both Panx1 knockout and control mice had similar and well defined expression of all epithelial markers (Fig 6A–6C). Furthermore, evaluation of the expression of luminal connexins [40] revealed a significant decrease in the relative number of Cx32 gap junction plaques, but not Cx26 or Cx30, in Panx1^-/-^ mice suggesting that Panx1^-/-^ mice may not have completed the full differentiation of the mammary gland (Fig 7). Taken together, Panx1^-/-^ mice have impaired mammary gland alveolar development during early lactation but relatively normal mammary gland differentiation.

### Lactating Panx1^-/-^ mice have normal mammary gland function

**Fig 8:**
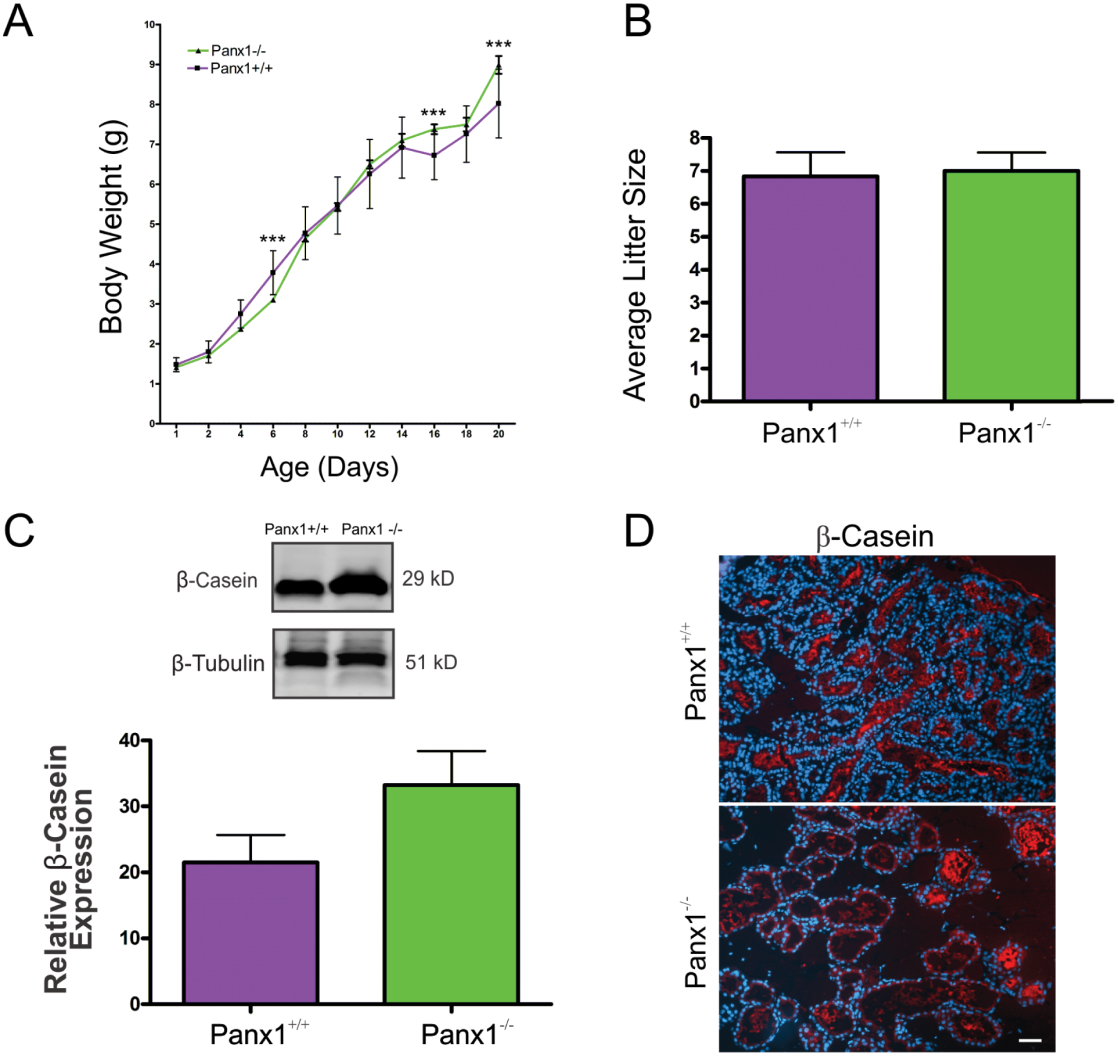
Panx1^-/-^ dams lactate and deliver milk to pups. (A) Evaluation of pup body weights from 1–20 day old pups revealed significant differences between Panx1^-/-^ mice at only a few ages (D6, D16 and D20) compared to Panx1^+/+^ mice (N = 30–50 pups). ***p≤0.001. (B) Litters from 10 Panx1^-/-^ dams were evaluated for litter sizes revealing similar numbers compared to control mice. (C, D) Western blot and immunofluorescent analysis of lactating mammary glands revealed no significant difference in the milk protein β-casein (red) in knockout and control mice. N = 6. Hoescht (blue) denotes nuclei. Values are means ± SEM.

We next determined whether the developmental defects associated with the mammary gland in lactating Panx1^-/-^ dams resulted in impaired feeding of pups (Fig 8A). Upon evaluating 30–50 pups from multiple litters, significantly decreased pup weights were only found at day 6 while significantly increased pup weights were recorded at day 18 and day 20 from Panx1 knockout dams compared to Panx1^+/+^ dams (Fig 8A). Importantly, Panx1^-/-^ dams had similar litter sizes to Panx1^+/+^ dams and pup death was uncommon (Fig 8B). Finally, western blot and immunofluorescent analysis of the common milk protein b-casein revealed no-significant difference between control and mutant mice suggesting milk production is unaffected (Fig 8C and 8D). Taken together, loss of Panx1 does not severely impair mammary gland function.

### Panx1^-/-^ mice have normal mammary gland involution

**Fig 9:**
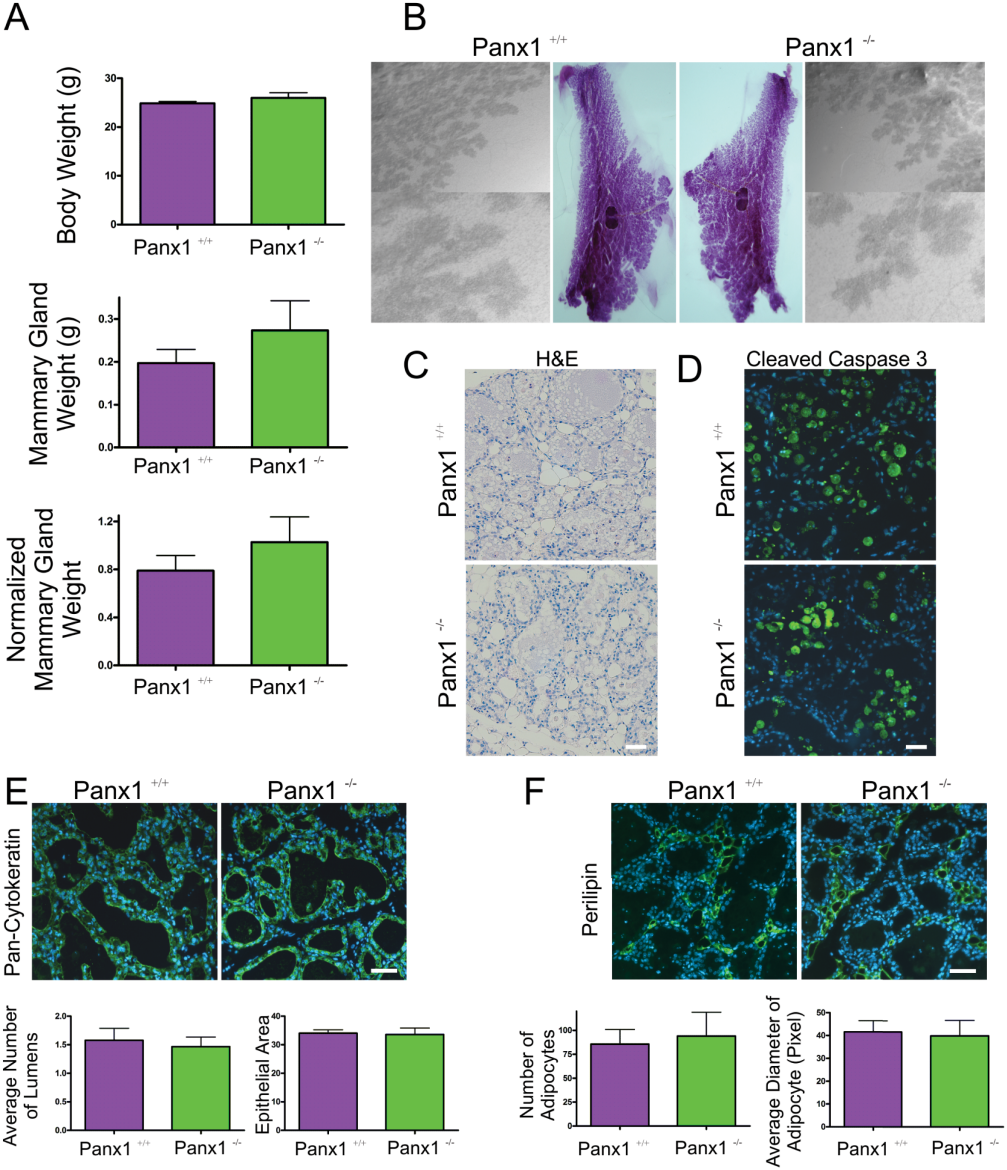
Involuting Panx1^-/-^ mouse mammary glands have normal gland regression. (A) Evaluation of body weight, mammary gland weight and normalized mammary gland weight revealed no significant difference between in Panx1^-/-^ mice compared to control mice. (B, C) Whole Mount and histological evaluation with haematoxylin and eosin revealed similar regression of glands in Panx1^-/-^ mice and Panx1^+/+^ mice. (D) Immunofluorescent analysis of the apoptotic marker cleaved caspase-3 revealed qualitatively similar amounts of apoptosis in Panx1 null mice and wild-type mice. (E) Quantification of the average epithelial area and alveolar lumen number as assessed with pan-cytokeratin (green) were similar in Panx1^-/-^ mice compared with control mice. (F) Quantification of the average number and diameter of adipocytes as assessed with perilipin (green) revealed similar numbers in the mammary gland of Panx1^-/-^ mice compared with control mice. Hoescht (blue) denotes nuclei. Values are means ± SEM. N = 5. Scale bars = 50 um.

In order to assess the role of Panx1 during involution, pups from Panx1^-/-^ and wild-type dams were force weaned at day 15 of lactation and three days later mammary glands were collected. Body weights, mammary gland weight and normalized mammary gland weight were similar between Panx1^-/-^ and control mice (Fig 9A). Whole mount and histological analysis revealed similar tissue architecture of Panx1^-/-^ mice compared to control mice (Fig 9B and 9C). Qualitative assessment of immunolabelled mammary gland for the apoptotic marker cleaved caspase 3 revealed comparably similar amount of cell death between Panx1^-/-^ mice and control mice (Fig 9D). Quantitative assessment of the number of alveolar lumen and relative epithelial area following labelling with pan-cytokeratin antibody also revealed a similar extent of epithelium in Panx1 knockout and wild-type involuting mammary glands suggesting comparable gland remodeling (Fig 9E). Similarly, evaluation of the number of adipocytes and the average diameter of adipocytes revealed no significant difference in the mammary glands of Panx1 knockout and control mice suggesting similar adipocyte repopulation of gland during involution (Fig 9F). Taken together, mammary glands of Panx1 null mice have comparable mammary gland involution to Panx1 wild-type mice.

### Panx1 expression is correlated with poor overall survival

**Fig 10:**
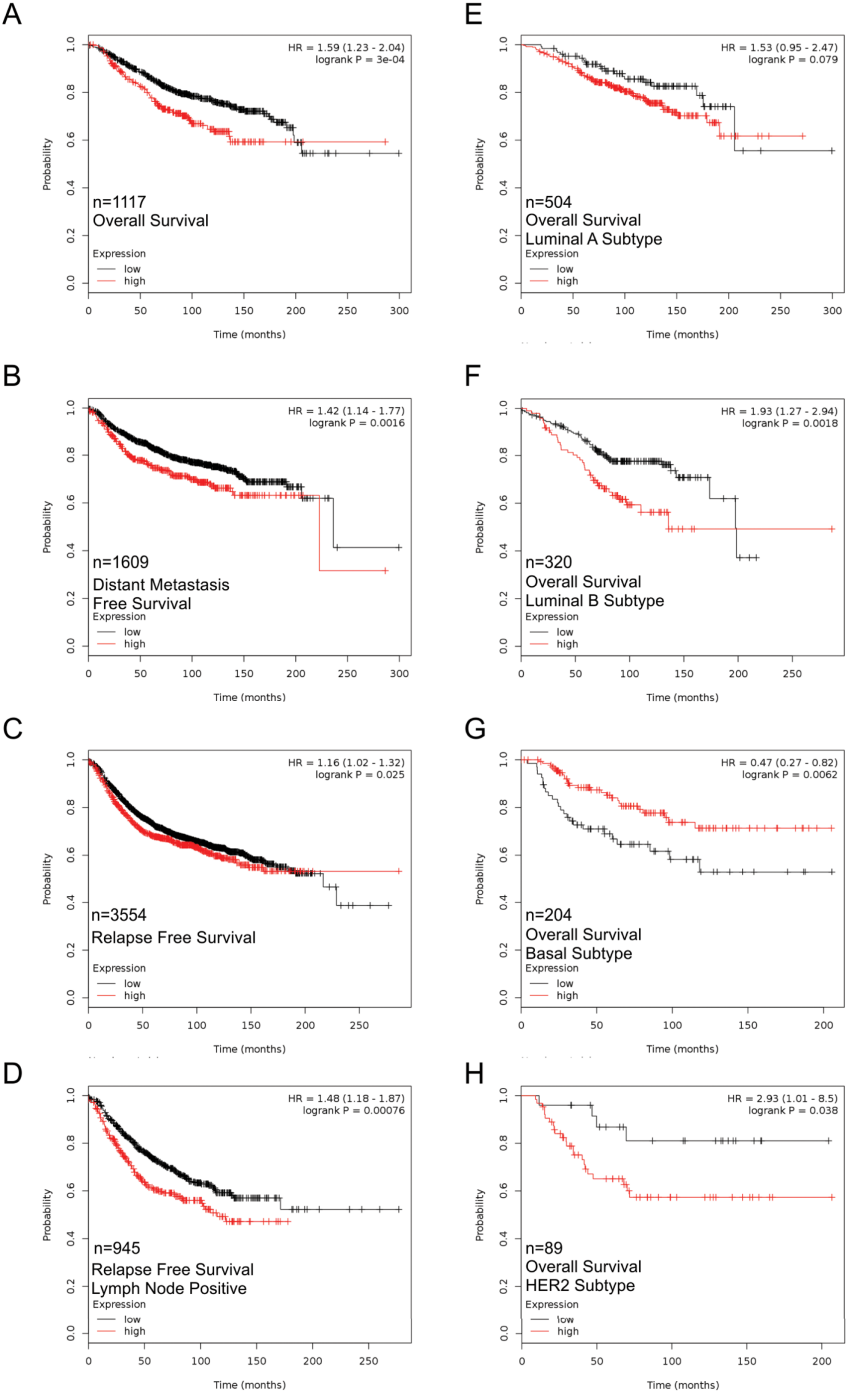
High PANX1 mRNA expression is correlated with poor overall survival, particularly in the Luminal B and Her2+ subtypes of breast cancer, as revealed by *in silico* analysis. The Kaplan-Meier Plotter was used to correlate PANX1 levels with patient survival. Breast cancer patient samples expressing high PANX1 mRNA expression were significantly correlated with worse overall survival (A), distant metastasis free survival (B), relapse free survival (C) and relapse free survival of lymph node positive patients (D) compared to patients with tumors expressing low levels of PANX1. (E) Tumors of the luminal A subtype had a similar correlation to overall survival in high and low PANX1 expressing samples. High PANX1 expression in luminal B (F) and Her2+ (H) tumors was associated with significantly worse overall survival compared to luminal B and Her2+ tumors expressing low levels of PANX1. Alternatively, high PANX1 expression in tumors of the basal subtype was associated with improved overall survival compared to tumors expressing low levels of PANX1 (G). A p-value less than 0.05 was considered significant.

Since Panx1 appeared to be a regulator of cell growth during early lactation we hypothesized that Panx1 levels may be relevant in breast cancer. Thus, PANX1 mRNA levels in breast tumors was correlated with clinical outcomes. Interestingly, high expression of PANX1 in patient tumors was significantly correlated with worse overall survival (OS), distant metastasis free survival (DMFS) and relapse free survival (RFS) compared with those that had low PANX1 expression (Fig 10A–10C). Importantly, when comparing PANX1 expression to OS in lymph node positive patients with advanced disease, PANX1 mRNA expression maintained a similar significant negative correlation with OS (Fig 10A–10C). In addition, PANX1 expression was compared with OS in the context of the Luminal A, Luminal B, Basal and Her2+ molecular subtypes. PANX1 expression was not significantly associated with OS in the luminal A subgroup (Fig 10E). However, PANX1 was significantly correlated with worse OS in luminal B and Her2+ samples (Fig 10F and 10H). Interestingly, PANX1 was associated with significantly better OS in tumors of the basal subtype suggesting differential roles of Panx1 that are dependent on the molecular subtype of the tumor (Fig 10G).

## Discussion

The purpose of this study was threefold; first, to assess whether Panx1 was expressed and dynamically regulated in the mammary gland; second, to determine whether loss of Panx1 altered the development of pubertal and lactating mice while establishing if Panx1 affected normal mammary gland function; and finally, to assess whether Panx1 in the mammary gland may have implications extending to patients with breast cancer.

### Panx1 is dynamically expressed in the mammary gland

Panx1 has an ubiquitous expression profile and has been reported in the mouse mammary gland based on expression profiling arrays in NCBI’s gene expression Omnibus database (ID 1416379, 78225667 [17]). In the murine mammary gland, Panx1 is expressed and upregulated during pregnancy where it remains elevated during lactation. Developmental regulation of Panx1 is associated with higher expression at earlier stages of development in many organs including the neonatal rat brain and murine newborn skin compared to aged counterparts [14,16,24]. Importantly, primary human muscle myoblasts induced to differentiate in culture upregulate the expression of Panx1 while ectopic expression of Panx1 in these cells induces differentiation *in vitro* [15]. Collectively, these results suggest a critical role for Panx1 in cell differentiation [15]. Unlike other organs, the mammary gland develops only a rudimentary ductal structure in prenatal mice and requires the onset of pregnancy to induce terminal differentiation of the gland [1]. Therefore, expression of Panx1 during pregnancy and lactation builds on the idea that Panx1 is upregulated in organs undergoing development and differentiation. As such, it might be expected that Panx1 is expressed in the embryonic mammary gland. While we cannot rule this out, loss of Panx1 does not significantly impair the ability of the gland to develop a rudimentary ductal structure and undergo normal ductal development during puberty in the virgin mammary gland. As a result, Panx1 may be more important in the pregnant than the embryonic mammary gland. This is similar to the gap junction, channel protein, Cx26, which has a critical role after the onset of pregnancy while being less important at earlier stages of mammary gland development [41]. Our results suggest that Panx1 is expressed mainly in luminal epithelial cells although many other cell types that can be found within the stromal compartment of the mammary gland have been reported to express Panx1 including fibroblasts [24], adipocytes [42], immune cells [43], erythrocytes [20], and cells of the vasculature [44]. While our staining was much more evident in the epithelium than in the stromal compartment of the gland, we have not ruled-out that Panx1 upregulation may also occur in these other cell type residents of the mammary gland. Importantly, the Panx1^-/-^ mice used in this study are null for Panx1 in all cell-types of the mammary gland.

### Panx1 is necessary for timely alveolar development and differentiation in the lactating mammary gland

Terminal development of the mammary gland is driven by hormonal signaling that regulates proliferation and differentiation in the mammary gland. Panx1^-/-^ mice had reduced alveolar development in early lactating mice due to impaired proliferation of the mammary gland that was not apparent at parturition. Day 2 and 3 of lactation represents a major proliferative time point of epithelial expansion in the mammary gland as indicated by increased DNA synthesis measured through recordings of [H^3^] thymidine incorporation [45,46]. Importantly, hormonal regulation driving epithelial cell proliferation during early lactation is believed to be due to pituitary prolactin and ovarian estrogen secretion [45]. Prolactin and estrogen have previously been shown to be absolutely essential to normal lobuloalveolar proliferation and differentiation as evidence by impaired lobuloalveolar defect in knockout mouse models of their respective receptors [40,47,48]. Interestingly, when mammary tissue from prolactin receptor knockout (PrlR^-/-^) mice is transplanted into wild-type cleared fat pads, due to issues of infertility, mammary gland proliferation and differentiation are impaired [48]. Of note, this coincides with complete loss of expression of the gap junction protein, Cx32 [48].

Similarly, estrogen receptor beta knockout (ERb^-/-^) mice also develop with impaired alveolar development and altered differentiation and have been further assessed to have reduced numbers of Cx32 gap junction plaques [40]. Therefore, it is interesting to speculate that Panx1 may impair either prolactin or estrogen mediated signaling as Panx1^-/-^ mice also present with mammary glands with fewer Cx32 gap junction plaques. It seems more likely that Panx1 is implicated in the ERb pathway, as ERb^-/-^ mice have reduced alveolar lumen number and present with dilated alveolar lumen similar to Panx1^-/-^ mice [40]. In addition, unlike PrlR^-/-^ mammary glands, loss of Panx1 or ERb^-/-^ mice is not associated with impaired milk production [48]. In fact, mammary function is relatively normal in Panx1^-/-^ mice, as pup weights of lactating dams were relatively unaffected similar to what has been seen in ERb^-/-^ mice. This suggests that impaired alveolar proliferation during early lactation in Panx1^-/-^ mice likely represents only a delay in the onset of proliferation during early lactation. However, differences do exist between the ERb^-/-^ and Panx1^-/-^ mice as mammary glands of ERb^-/-^ mice were associated with dysregulated epithelial markers E-cadherin and b-catenin which was not observed in Panx1^-/-^ mice. This suggests that Panx1 is downstream of ERb signaling, as ERb^-/-^ mice have a more severe phenotype. Taken together, we propose that Panx1 ablation may impair ERβ rather than PrlR signaling. Alternatively, since Panx1 has been reported to be expressed in the pituitary gland and the ovary, we cannot fully rule-out that loss of Panx1 in these organs may be mediating alveolar defects in the mammary gland [13,49,50]. However, this seems unlikely as Panx1^-/-^ mice have a relatively normal phenotype, which might be expected to be more severe if hormonal signaling was dramatically impaired.

While the mechanism of how Panx1 acts in the mammary gland is unknown, most studies assessing the role of Panx1 have found that Panx1 channel function involves ATP release that acts through purinergic receptors [51]. Interestingly, ATP release has been demonstrated to be important in Ca^2+^ wave propagation in coordination with P2Y and P2X receptors [52]. Intriguingly, mechanical stimulation of mammary tumor cell leads to the release of nucleotides through an unknown mechanism that induces Ca^2+^ signaling [53]. It remains interesting to speculate that Panx1 plays a role in this process. Importantly, Ca^2+^ signaling is extremely important in the lactating mammary gland and contributes to proliferation, secretion, and myoepithelial contraction [54]. However, this remains to be verified by future studies.

### Loss of Panx1 does not impair involution

In an earlier report, Panx1 was implicated as a critical channel during cell apoptosis, in which activation of caspases led to truncation of the C-terminal tail of Panx1 and the release of nucleotides that act as “find-me” signals for phagocytic cell-mediated clearance [19]. We predicted that loss of Panx1 would impede normal mammary gland involution in which extensive apoptosis occurs requiring epithelial cell clearance from the mammary gland. However, loss of Panx1 did not affect mammary gland involution based on our assessment of epithelial cell area or adipocyte repopulation of the gland 72 hours following force weaning of the pups. Interestingly, Monk et al. has shown that apoptotic clearance in the mammary gland is mediated almost exclusively by alveolar epithelial cells, as opposed to macrophages [55,56]. This suggests that Panx1 is not the channel linked to cell clearance by which “find me” signals are released by apoptotic mammary epithelial cells, or that the loss of Panx1 channels is compensated by other nucleotide-releasing channels, or there is another mechanism involved [57]. Collectively, Panx1 appears dispensable for normal murine mammary gland involution at least in the first three days following forced weaning. However, while we have no evidence that Panx2 or Panx3 are upregulated in the mammary gland upon Panx1 ablation, it remains possible that baseline levels of Panx2 or Panx3 may act to preserve gland function.

### Panx1 in breast cancer

High PANX1 expression was correlated with worse OS, RFS and DMFS in breast tumors from patients suggesting that PANX1 may act as a tumor facilitator in breast cancer. This is supported by Furlow et al. who demonstrated that PANX1 channel activity was critical in promoting breast cancer lung metastasis by increasing metastatic cell survival during extravasation [29]. Interestingly, the effect of PANX1 was similar in breast cancer cells of the basal (MDA-MB-468) and claudin-low molecular subtype (MDA-MB-231, BT-549), suggesting that this novel role for PANX1 may be seen in multiple breast cancer subtypes [29,58,59]. While we did not compare the correlation between PANX1 expression and clinical outcomes in the claudin-low subtype, we found that high PANX1 expression was correlated with better overall survival in the basal subtype group in contrast to those in MDA-MB-468 cells seen by Furlow et al [29]. Though these results do not make for the best comparison, these differential findings in the basal subgroup may speak to a dual role for PANX1 in tumorigenesis. Indeed, despite relatively few studies assessing the role of PANX1 in cancer, rodent Panx1 and/or human PANX1 have already been implicated as both a tumor suppressor and tumor facilitator in different types of cancer [30–32]. This suggests that the role of PANX1 in tumorigenesis may be complex and dependent on tumor type and stage of the disease. Nevertheless, the results to date suggest a tumor facilitating role of PANX1 in breast cancer. It remains interesting to speculate that PANX1’s role in regulating cell proliferation in normal development may translate into dysregulated growth in the context of the primary tumor. Regardless, there is a need for further studies on the role of PANX1 in breast cancer.

In summary, through the use of a global Panx1 knockout mouse, it is clear that Panx1 is not critical for the normal function of the gland but is necessary for timely alveolar development and proliferation following the transition of the mammary gland from pregnancy into early lactation. Importantly, PANX1 expression within the mammary gland may have important implications to patients with breast cancer where increased expression of PANX1 is generally correlated with a worse clinical outcome.
